# Elaboration versus Suppression of Cued Memories: Influence of Memory Recall Instruction and Success on Parietal Lobe, Default Network, and Hippocampal Activity

**DOI:** 10.1371/journal.pone.0089037

**Published:** 2014-02-19

**Authors:** Sarah I. Gimbel, James B. Brewer

**Affiliations:** 1 Department of Neurosciences, University of California San Diego, San Diego, California, United States of America; 2 Department of Radiology, University of California San Diego, San Diego, California, United States of America; University of Texas at Dallas, United States of America

## Abstract

Functional imaging studies of episodic memory retrieval consistently report task-evoked and memory-related activity in the medial temporal lobe, default network and parietal lobe subregions. Associated components of memory retrieval, such as attention-shifts, search, retrieval success, and post-retrieval processing also influence regional activity, but these influences remain ill-defined. To better understand how top-down control affects the neural bases of memory retrieval, we examined how regional activity responses were modulated by task goals during recall success or failure. Specifically, activity was examined during memory suppression, recall, and elaborative recall of paired-associates. Parietal lobe was subdivided into dorsal (BA 7), posterior ventral (BA 39), and anterior ventral (BA 40) regions, which were investigated separately to examine hypothesized distinctions in sub-regional functional responses related to differential attention-to-memory and memory strength. Top-down suppression of recall abolished memory strength effects in BA 39, which showed a task-negative response, and BA 40, which showed a task-positive response. The task-negative response in default network showed greater negatively-deflected signal for forgotten pairs when task goals required recall. Hippocampal activity was task-positive and was influenced by memory strength only when task goals required recall. As in previous studies, we show a memory strength effect in parietal lobe and hippocampus, but we show that this effect is top-down controlled and sensitive to whether the subject is trying to suppress or retrieve a memory. These regions are all implicated in memory recall, but their individual activity patterns show distinct memory-strength-related responses when task goals are varied. In parietal lobe, default network, and hippocampus, top-down control can override the commonly identified effects of memory strength.

## Introduction

The retrieval of episodic memories is elemental to nearly all aspects of everyday life, yet little is known about how the brain performs and integrates the component processes of memory retrieval. Some brain functions might be called upon specifically for episodic memory retrieval, while other functions, also perhaps critical for memory retrieval, are broadly involved in a range of cognitive processes. Imaging and electrophysiological studies have sought to link regional brain activity to episodic memory function and have demonstrated that activity in the human hippocampus is modulated during both encoding and retrieval of memories [1]. Further, these studies have provided evidence supporting top-down modulation of hippocampal activity during memory retrieval and suppression. The role of the parietal lobe in episodic memory retrieval is controversial, and interpretation is complicated by the fact that this region includes several subregions with distinct functions, functional responses, and connectivity [2]. Electrophysiological studies suggest that regions of the parietal lobe are engaged prior to recall of a memory [3], supporting that these regions are driven by direction of attention toward memory [4]–[6]. The present study examines how task goals modulate regional activity during memory recall and additionally modulate memory strength effects. Given what is already known about regional contributions to these tasks, the analysis will focus on subregions of the parietal lobe, medial temporal lobe, and default network regions. By identifying how recall-related brain activity is influenced by task goals, or top-down processes, this work is a step toward understanding how task instruction and recall success modulate activity in human medial temporal lobe, parietal lobe and default network regions.

The parietal lobe has been implicated in a wide variety of tasks, and studies of memory retrieval have reported relatively increased blood oxygen level dependent (BOLD) activity in this region for successful retrieval versus comparison trials [4], [5], [7]–[11]. In general, strong memories tend to show relatively greater activity in the precuneus, lateral parietal cortex/intraparietal sulcus, retrosplenial cortex, and posterior cingulate than weak memories [6]–[9], [11], [12]. Modulation of parietal subregional activity during memory retrieval and effects of parietal lobe lesions on memory has led to distinct hypotheses about the role of the parietal lobe in recognition of a presented stimulus and recollection of associated information. One hypothesis supports that the parietal lobe holds information until a critical threshold is reached for memory recognition, thus acting as a mnemonic accumulator [6], [13] or buffer [6], [11], [14]. Another hypothesis indicates the role of the parietal lobe as contributing to the subjective experience of recollection [15]–[17]. A third hypothesis is that the lateral parietal cortex is modulated by direction of attention away from external stimuli and toward internal memory representations [3]–[6]. The direct comparison of memory retrieval (and ruminating on the contents of retrieval) versus memory suppression can allow insight into whether regional parietal involvement is related to the experience of recollection or, instead, related to broader attentional aspects of the task. Further, examination of instances of retrieval failure or success under these conditions can determine whether accurate retrieval influences the region's activity, shedding additional light on the interaction between attention and memory in the parietal lobe. Separable subregions of the parietal lobe serve different functions in the performance of memory tasks [18], highlighting the need for studies that probe top-down modulation of the commonly reported retrieval activations.

In discussing memory retrieval, the parietal lobe is often subdivided into dorsal parietal cortex and ventral parietal cortex, with differing theories on the exact contributions of each subregion. One view, building on the attention to memory hypothesis, suggests that the dorsal parietal cortex reflects top-down direction of attention to retrieval, or goal-driven attention [4], [5]. This region shows increased activation for low-confidence memory judgments, in which subjects need greater attention to memory retrieval [19]–[21]. In contrast, the ventral parietal cortex is thought to play a more direct role in memory retrieval that is not influenced by top-down processes. This region shows sensitivity to the frequencies of old and new items [11], [22], and some have suggested that this differential activity reflects the spontaneous capture of attention for old items, a bottom-up attention process [4], [5]. Further complicating the interpretation of parietal lobe activity is its regional overlap with the default network, a set of regions more active in the resting state than during the performance of tasks requiring external focus of attention [23]. Since part of the ventral parietal cortex falls within this network of regions, default network activity should be taken into consideration when examining ventral parietal cortex activation [24].

In addition to parietal activations related to memory, the default network is known to show modulations in activation during tasks of memory retrieval. Default network activity has been attributed to mind wandering [25] and internally directed thought [23], [26]–[29]. Many studies have shown that default network activity decreases during attention-demanding cognitive tasks [23]. A common interpretation is that default network activity is related to spontaneous, task-related, self-referential, or introspective mental activity [26] and general information gathering and evaluation [23]. The default network is deactivated during more difficult tasks requiring external focus of attention [30] and activated during tasks of memory recognition, which require more internally directed thought [24]. These tasks of memory recognition, however, are often easier and faster than the selected comparison task [31]. Some studies have focused on task-induced deactivation, and found that there is increased task-induced deactivation with increased task difficulty [27]. Taken together, these findings raise important questions about the basis for differential activity of default network during successful and unsuccessful memory retrieval. Specifically, there is a question of whether this difference commonly observed in default network activity is due to increased activity during successful memory retrieval or decreased activity during unsuccessful retrieval, which demands greater search and is a more difficult task.

**Figure 1:**
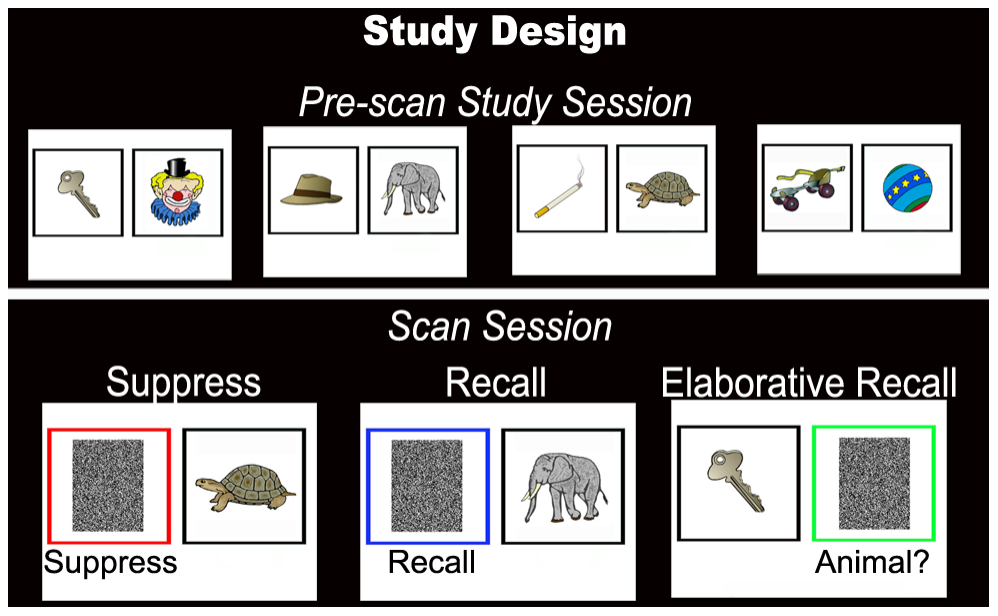
Study design. Each subject participated in the pre-scan study session outside of the scanner. Subjects were asked to memorize 60 pairs of images that were learned to 100% criterion. During scanning, subjects performed the event-related task in which they were asked to either ‘suppress,’ ‘recall,’ or ‘elaboratively recall’ the pair of the item presented. Trials were interleaved with a jittered fixation cross baseline.

In the present study, effects of task instruction were explored to identify how top-down processes and retrieval success modulate activity in commonly identified retrieval activations in parietal lobe, default network, and hippocampus. Subjects viewed 60 pairs of color images in a pre-scan study session. During scanning, subjects saw an item from a studied pair along with one of the following instructions: 1) suppress its pair, 2) recall its pair, or 3) recall the pair and answer a question about the recalled item (Figure 1). In a post-scan test, subjects were again shown one item from each pair and asked to verbally identify the image with which it had been paired. Using the post-scan test results, pairs were classified as being remembered (during the post-scan test) or forgotten (during the post-scan test) for further exploration and analysis. This post-scan test was used as a gauge of memory recall for the stimulus cue and its missing pair shown during the scanned task without inserting potentially contaminating meta-memory judgments while recording brain activity. By examining suppression, recall, and elaborative recall of remembered and forgotten associative memories we sought to disentangle the differential influences of task difficulty, memory strength, and introspection on activations linked to memory in the parietal lobe, default network, and hippocampus.

Based on the literature described above, hypotheses to inform the ongoing debate about regional brain involvement in episodic memory retrieval were generated. Separate hypotheses were tested for anatomically and functionally delineated brain regions: 1) If dorsal parietal regions are influenced by top-down attention to memory, then instruction to recall an item's pair should result in greater dorsal parietal activation than instruction to suppress an item's pair. Further, instruction to recall and elaborate on the memory (by performing a classification of the recalled object) should result in still greater dorsal parietal activity, but only in trials where recall is successful. In trials where recall fails, elaborative recall and simple recall should elicit qualitatively similar activity, given that such trials should lead only to search processes, whereas success is required prior to rumination on the products of retrieval or post-retrieval elaboration of the memory. Instruction to suppress an item's pair should yield no activity differences across remembered and forgotten pairs, given that top-down attention to memory (and search) should be absent and therefore balanced. If such a pattern is noted in the dorsal parietal lobe, this would support the attention-to-memory hypothesis of dorsal parietal lobe function. 2) If ventral parietal regions are influenced by bottom-up processes, then instruction to suppress, recall, or recall with elaboration should have minimal influence on brain activity. The primary influence on brain activity in these regions should be the success or failure of recall under instructions to retrieve, and, perhaps, absence of recall under instruction to suppress. If such a pattern is noted in the ventral parietal lobe, it would support the dual-process hypothesis of parietal function. 3) Default network is expected to be most influenced by task difficulty; thus, it should be maximally suppressed during elaborative recall and during failure of recall under instructions to retrieve. 4) If hippocampal activity can be modulated in a top-down fashion as prior studies suggest, then successful recall under instruction to recall should result in increased hippocampal activity relative to conditions without instruction to recall.

## Materials and Methods

### Subjects

Twelve healthy, right-handed subjects were recruited from the University of California, San Diego community and the surrounding area (mean age = 27±3 years, 8 male). Subjects had normal or corrected-to-normal vision and no history of neurological of psychiatric disorders.

### Ethics Statement

Each subject gave written informed consent and was paid $40 for participation. The study procedures and the written consent were approved by the Institutional Review Board of the Human Research Protections Program at the University of California, San Diego.

### Stimuli

Stimuli were 120 color drawings of common objects selected from Rossion and Pourtois color Snodgrass images [32] randomly paired into 60 pairs.

### Experimental Design

In a pre-scan study session, subjects were exposed to each of the 60 pairs until the pairs were learned to criterion. Subjects studied all 60 pairs, and then were tested on each pair. Unlearned pairs were shown again until each pair could be correctly identified during testing. During scanning, subjects were presented with two adjacent noise-mask-filled boxes on the viewing screen, one outlined in black and one outlined in either red, blue, or green (Figure 1). After 1 second, an image from the studied pairs appeared in the black box for 0.5 seconds. Subjects were asked to view the item and to either (1) suppress the item that had been paired with the viewed item (suppress, red box), (2) recall the pair of the viewed item (recall, blue box), or (3) recall the pair of the viewed item and answer a presented ‘yes/no’ question about the recalled item (elaborative recall, green box). The question that appeared with the elaborative recall trial was varied to prevent prediction of the question. Trials were jittered with 0, 1.5, 3, or 4.5 seconds of fixation baseline to optimize the study design [33]. Each subject underwent a single scan session that included five 428 second scans. Each run included one presentation of each pair, for a total of five presentations of each pair throughout the study. Instructions stayed consistent for each presentation of the same pair (a single pair was always suppressed, recalled, or recalled with elaboration). Assignment of pairs to trial types was counterbalanced across subjects. Since verbal responses of recollection could not be collected during scanning and the use of meta-memory judgments was avoided, a post-scan test was given in which subjects saw one item from each pair of images and verbally recollected the item paired with the presented item. Pairs correctly recalled were used in subsequent analyses as ‘remembered’ and pairs not correctly recalled were deemed ‘forgotten.’ Though uncertainty remains that pairs forgotten in the post-scan test were also forgotten during the scan, this post-scan surrogate for task performance and memory strength judgments was selected to avoid contaminating the scan session with unwanted cognitive processing and the associated influences of metamemory judgments on brain activity, given temporal linkage to the retrieval event. Subject report after the scan session confirmed that they were indeed performing the recall and suppress tasks as instructed.

### Functional MRI Parameters

Imaging was conducted in a 3T GE scanner at the Keck Center for Functional MRI at the University of California, San Diego. Functional images were acquired using a gradient echo echo-planar, T2*-weighted pulse sequence (repetition time = 1.5 s, one shot per repetition, echo time = 30, flip angle = 90°, bandwidth = 31.25 MHz). Twenty-two slices covering the entire brain were acquired perpendicular to the long axis of the hippocampus with 4×4×7 mm voxels, allowing greater summation of activity along the hippocampal axial plane [34]. A T1-weighted high resolution (1×1×1 mm), three-dimensional fast spoiled gradient recalled anatomical dataset was collected. A structural scan was acquired in the same slice locations as the functional images for use in confirming alignment of functional data to the high-resolution anatomical scan.

### Data Analysis

Data from each run were reconstructed and then field map corrected [35]. Slices were temporally aligned and co-registered with a 3D registration algorithm. Voxels outside the brain were removed using a threshold mask of the functional data. Functional runs were corrected for motion. A general linear model was constructed using multiple regression analysis and included six motion regressors from the registration process and regressors for ‘suppress,’ ‘recall,’ and ‘elaborative recall’ condition ‘remembered’ and ‘forgotten’ memory responses. A second general linear model was constructed using regressors for the presence and absence of an active task. Standard landmarks (anterior and posterior commissures) were defined manually on the anatomical scans, and then the anatomical and functional scans were transformed into Talairach space [36] using AFNI nearest neighbor interpolation [37].

In order to improve alignment of medial temporal lobe structures, the region of interest large deformation diffeomorphic metric mapping (ROI-LDDMM) alignment technique [38] was used. First, the hippocampus, entorhinal cortex, perirhinal cortex, and parahippocampal cortex were hand drawn for each subject. Entorhinal and perirhinal cortices were defined according to the landmarks used by Insausti et al. [39]; the caudal border of the perirhinal cortex was defined as 3 mm caudal to the disappearance of the uncus, and the parahippocampal cortex was defined as the portion of parahippocampal gyrus caudal to the perirhinal cortex and rostral to the splenium of the corpus callosum [39]. Anatomical regions of interest for each subject were normalized to a previously defined template using ROI-LDDMM [40] and the transformation was also applied to individual functional data to ensure alignment with the anatomical structures.

A hemodynamic response function was estimated for the 15 seconds following the onset of the stimulus using signal deconvolution. Voxel-wise t-tests (two-tailed) were performed to compare average BOLD signal between conditions. After individual deconvolution analysis, single-subject parameter estimates were entered into group level analyses. Voxel-wise two-tailed *t*-tests (planned comparisons) and ANOVAs were performed within each region of interest to compare average area under the curve between conditions and between the presence and absence of a task. All reported results have been corrected for multiple comparisons, where appropriate. In the default network analysis (task vs. no task) and in the contrast that identified the hippocampus (remembered vs. forgotten), clusters were defined with a connectivity of 4 mm between voxel-centers and included at least 5 voxels for a whole brain significance of *p*<.05 and a voxel-wise significance of *p*<.001 when corrected for multiple comparisons (using alpha probability simulations calculated with the AFNI plugin, AlphaSim). These clusters were displayed using a statistical map overlaid onto an average structural image of all 12 subjects; the average hemodynamic response function (beta value) was then extracted for each cluster of interest. In the task vs. no task identification of the default network, 16 clusters were defined in 10 known default network regions. These clusters showed common activation patterns, and were thus averaged together for display purposes. In the parietal lobe analysis, Brodmann areas were defined anatomically based on the Talairach atlas, and BA 7, 39, and 40 were used to extract the average hemodynamic response function for parietal lobe subregions.

## Results

### Behavioral Analysis

In the ‘elaborative recall’ trials, subjects responded in 96% of trials (average reaction time: 1.94 ±.45 seconds). These results are similar to other studies using this task [3], [41]. Of these trials, subjects made a correct classification in 75±1% of trials, an incorrect classification in 9±1% of trials, and were “unsure” in 16±1% of trials. Similarly, in the post-scan memory test, subjects correctly identified the pair of the presented image 75±3% of the time for pairs that had been recalled with elaboration, 74±4% for pairs that had been recalled, and 71±3% for pairs that had been suppressed. Since each pair was presented 5 times during testing, this means that on average, there were 71.6±3.4 trials in the suppress remembered bin, 28.4±3.4 trials in the suppress forgotten bin, 74.1±4.2 trials in the recall remembered bin, 25.9±4.2 trials in the recall forgotten bin, 75.3±2.9 trials in the elaborative recall remembered bin, and 24.7±2.9 trials in the elaborative recall forgotten bin. There was no difference in the percentage of pairs remembered for each condition in the post-scan test (*F_(1,10)_* = .288, *p* = .751, MSE = .020).

### fMRI Analysis - Parietal Lobe Response

**Figure 2:**
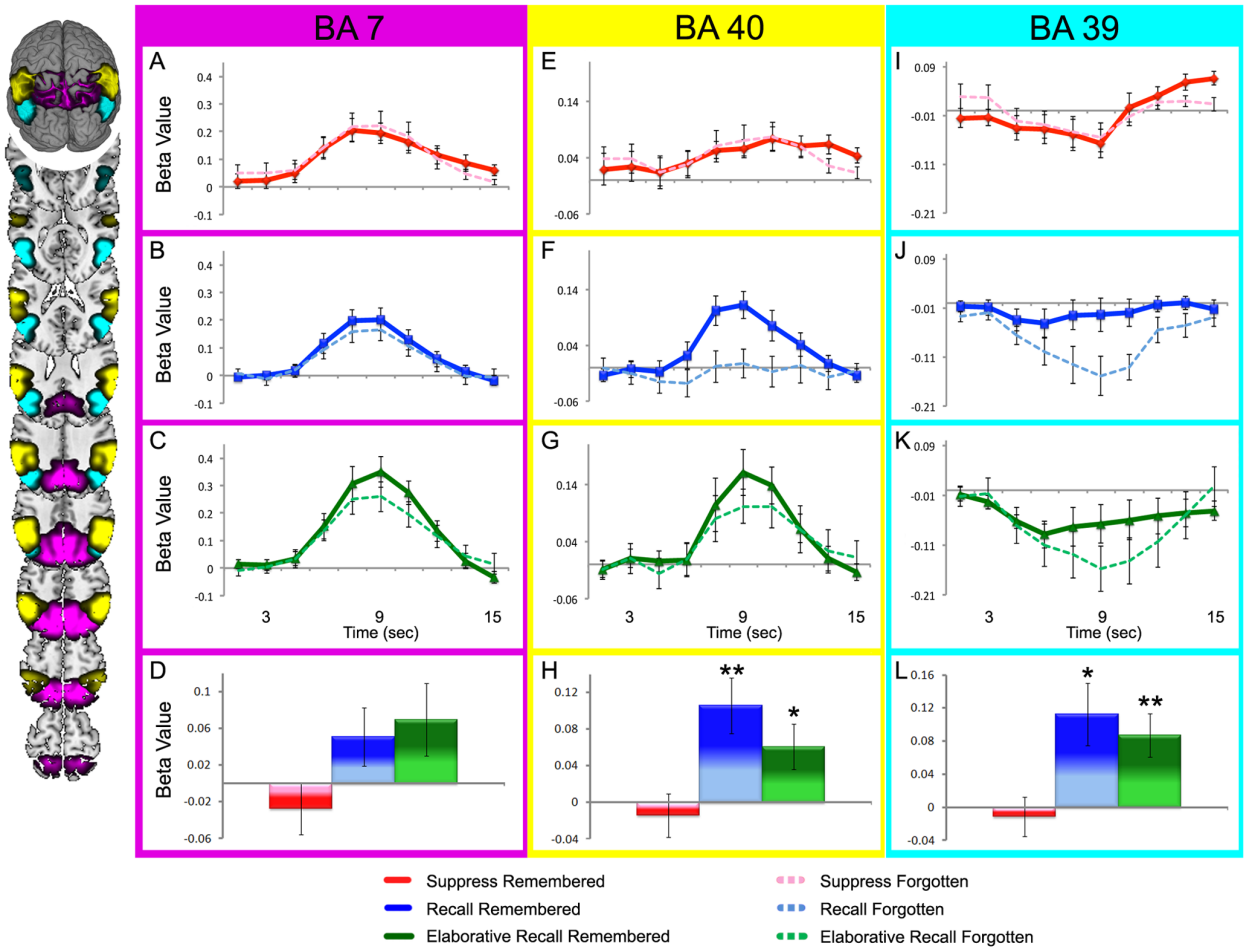
Activation during suppression, recall, and elaborative recall differs for subregions of the parietal lobe. Activation in Brodmann Areas 7 (magenta), 40 (yellow), and 39 (cyan). Impulse response curves for suppress (A, E, I), recall (B, F, J), and elaborative recall (C, G, K) remembered and forgotten trials and difference scores (D, H, L) representing the difference in activation for remembered trials versus forgotten trials plotted for suppress (red), recall (blue), and elaborative recall (green) trials. Impulse response curves and difference plots for the A–D) dorsal parietal (BA 7) E–H) anterior ventral parietal (BA 40), and I–L) posterior ventral parietal (BA 39) cortices. Brodmann Areas presented on a standard brain. Error bars represent standard error of the mean, * *p*<.05, ** *p*<.01.

Dorsal parietal cortex (BA7, Figure 2, pink) and anterior ventral parietal cortex (BA 40, Figure 2, cyan) showed task-positive activity (increased activity from baseline) during elaborative recall, recall, and suppress conditions. In contrast, posterior parietal cortex (BA 39) showed task-negative activity (decreased activity below baseline) during forgotten recall trials and during both remembered and forgotten elaborative recall trials (Figure 2, yellow).

All three regions showed a main effect of retrieval instruction (BA 7: *F_(1,10)_* = 7.993, *p*<.01; BA 40: *F_(1,10)_* = 4.071, *p*<.05; BA 39: *F_ (1,10)_* = 17.782, *p*<.01). While posterior parietal cortex showed this main effect of retrieval instruction, it was almost entirely driven by greater negative deflection for forgotten items when a recall attempt was made (Figure 2J,K). Additionally, there is a triple interaction between parietal subregion, trial type, and memory strength (*F_(1,10)_* = 9.231, *p*<.05, MSE = .133).

In all regions targeted in this study, differential brain activity for remembered and forgotten pairs was seen only under retrieval conditions and not during suppress conditions (Table 1). In dorsal parietal cortex (BA 7), there was no difference between remembered pairs and forgotten pairs during recall (*t_(11)_* = 1.606; *p* = .137, Figure 2B), elaborative recall (*t_(11)_* = 1.748; *p* = .108, Figure 2C), or suppress trials (*t_(11)_* = .953; *p* = .361, Figure 2A). Memory-strength-related activity was numerically greater for elaborative recall than for recall, with a trend toward significance (*F_(1,10)_* = 2.761; *p* = .078, MSE = .025). In this region, there was an interaction of trial type and memory strength (*F_(1,10)_* = 4.685; *p*<.05, MSE = .003).

**Table 1:** Influence of task on memory strength effects (t-value) in the parietal lobe, default network, and hippocampus.

	Parietal BA^4^ 7	Parietal BA^4^ 40	Parietal BA^4^ 39	Default Network	Hippocampus
**Memory effect – S1**	.953	.606	.488	.569	.207
**Memory effect – R2**	1.604	3.497**	3.004*	5.921***	3.624**
**Memory effect – ER3**	1.750	2.437*	3.306**	5.921***	2.059
**Direction of deflection**	↑	↑	↓	↓	↑

1 S – Suppress,

2 R – Recall,

3 ER – Elaborative Recall,

4 BA – Brodmann Area.

* *p*<.05,

** *p*<.01,

*** *p*<.001.

In anterior ventral parietal cortex (BA 40), there was an overall difference in remembered trials between elaborative recall, recall, and suppress conditions (*F_(1,10)_* = 3.879; *p*<.05, MSE = .009). In this region, activity for remembered trials was greater than for forgotten trials in the recall task (*t_(11)_* = 2.990; *p*<.05, Figure 2F) and elaborative recall task (*t_(11)_* = 3.307; *p<.01*, Figure 2G). Again, there was no memory-strength-related difference for suppress trials (*t_(11)_* = .488; *p* = .635, Figure 2E). In this region, there was a significant interaction of trial type and memory strength (*F_(1,10)_* = 5.034; *p*<.05, MSE = .004).

Posterior ventral parietal cortex (BA 39) was modulated by recall success, and showed activity differences between remembered and forgotten trials in the recall (*t_(11)_* = 3.455, *p*<.01, Figure 2J) and elaborative-recall conditions (*t_(11)_* = 2.431; *p*<.05, Figure 2K). In this region, the memory-strength-related differences were a result of decreased activity for forgotten pairs, as opposed to an increase in activity for remembered pairs. There was no memory-strength-related activity for suppress trials (*t_(11)_* = .606; *p* = .557, Figure 2I). For remembered trials, there was no difference between elaborative recall, recall, and suppress conditions in this region (*F_(1,10)_* = 0.616; *p* = .546, MSE = .013).There was, however, a main effect of memory strength (*F_(1,10)_* = 18.641, *p*<.01, MSE = .004) and an interaction of trial type and memory strength (*F_(1,10)_* 4.653; *p*<.05, MSE = .006).

### fMRI Analysis - Default Network Response

**Figure 3:**
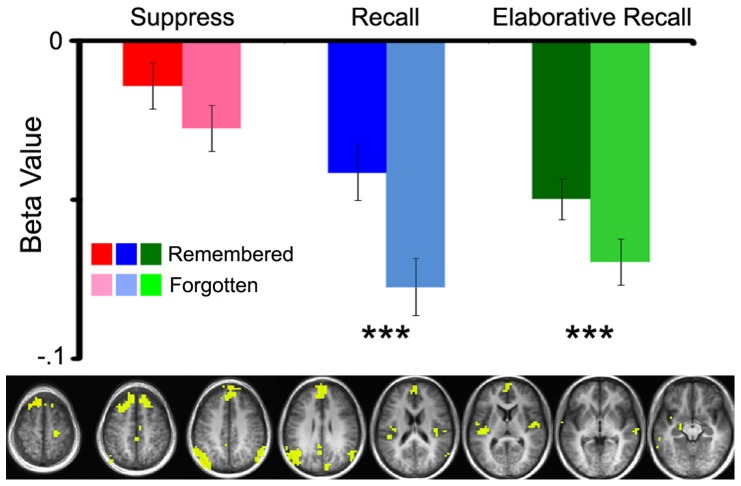
Task instruction and recall success influence the default network. *Below*, Activity identified in the default network (yellow), revealed by the task minus fixation contrast. Significant clusters (*p*<.01) used to extract average activity for each task condition, for both remembered and forgotten responses. Within the recall and elaborative recall conditions, there was greater suppression of activity for forgotten pairs compared to remembered pairs; *** *p*<.001. Activity presented on an average anatomical brain of all 12 study participants. Error bars represent standard error of the mean.

Sixteen clusters of activity in ten regions of the brain (superior frontal gyrus, medial frontal gyrus, insula, precentral gyrus, middle temporal gyrus, cingulate gyrus, precuneus, cuneus, inferior parietal gyrus, and superior temporal gyrus) were found to have less activity during the performance of a task than during fixation baseline (Figure 3). These regions, identified as part of the default network, commonly show a decrease in BOLD activity during an active task. Further analysis of the activity across this network of regions showed a greater decrease in activity for forgotten pair trials than for remembered pair trials in the recall (*t_(11)_* = 7.945; *p*<.001) and recall-classify tasks (*t_(11)_* = 3.943; *p*<.001), but not in the suppress task (*t_(11)_* = .569; *p* = .581). There was a main effect of memory strength in this set of regions (*F_(1,10)_* = 7.724, *p*<.05, MSE = .003).

### fMRI Analysis - Hippocampal Response

**Figure 4:**
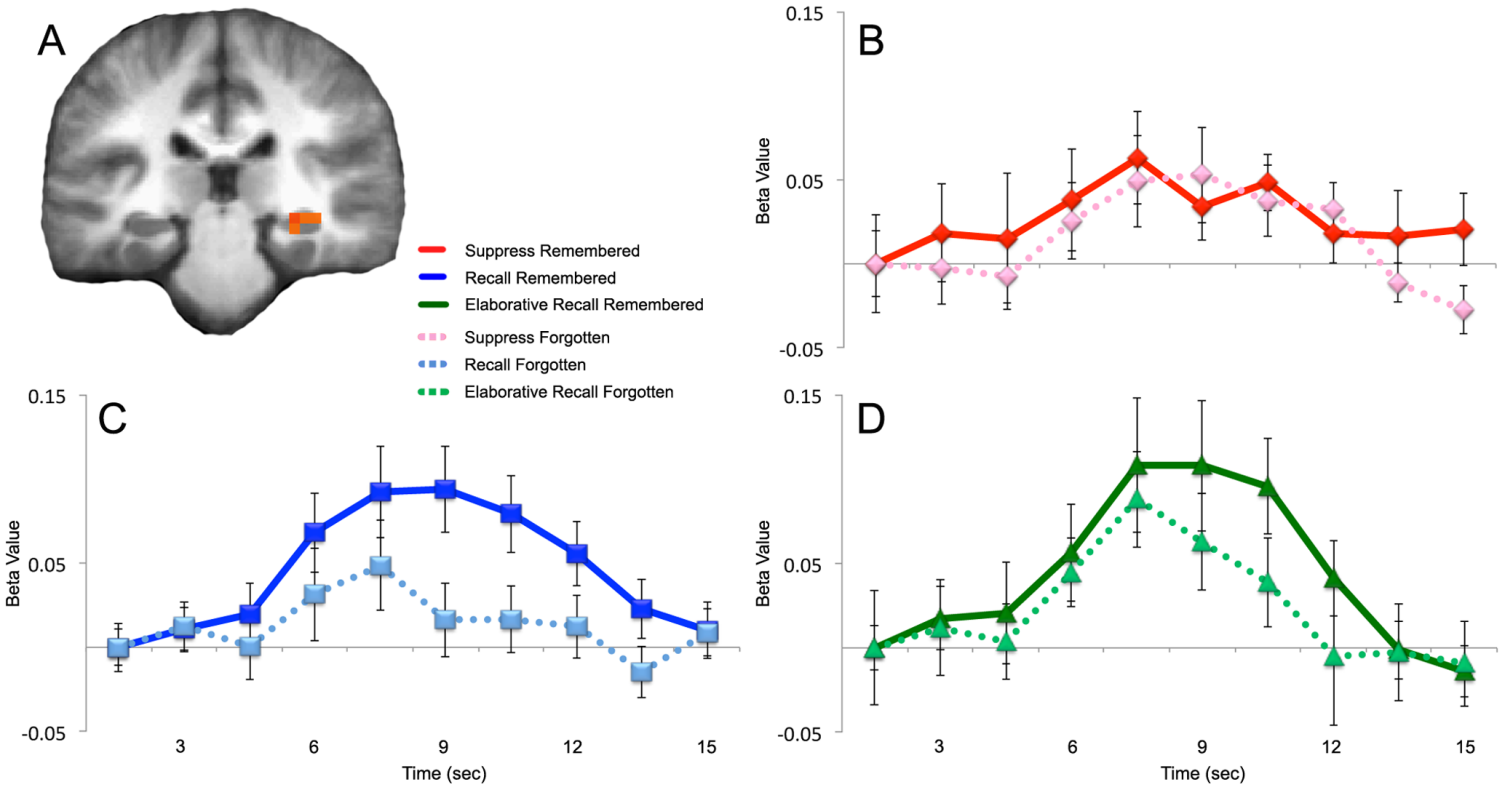
Hippocampal activity during ‘suppress,’ ‘recall,’ and ‘elaborative recall’ remembered and forgotten trials. A) A cluster map of remembered minus forgotten trials (*p*<.01) was overlaid on an average anatomical brain from all twelve subjects. B–D) BOLD activity was increased in the hippocampus for trials where the subject was instructed to recall the previously studied pair and successfully did so (C,D), but not when there was no instruction to recall (B). Right posterior hippocampal cluster centered at (30, -26, -4). Activity presented on an average anatomical brain of all 12 study participants. Error bars represent standard error of the mean.

Comparing remembered trials to forgotten trials, each presented under instructions to recall, right posterior hippocampus was the single region of significance across the brain at the threshold of *p*<.01, corrected for multiple comparisons (Figure 4A). The hippocampus, which traditionally is identified as part of the default network, was influenced by recall success and the explicit instruction to recall. There were main effects of retrieval instruction (*F_(1,10)_* = 7.993, *p*<.01, MSE = .002) and memory strength (*F_(1,10)_* = 12.333, *p*<.01, MSE = .004). Forgotten trials elicited no detectable hippocampal response, while remembered trials elicited a task-positive impulse response (Figure 4C,D). Under instruction to suppress retrieval, neither remembered nor forgotten trials elicited a hippocampal response that differed from baseline (Figure 4B). As such, the hippocampus responded with an increase in activity only for trials in which subjects were instructed to recall and successfully did so, resulting in a trend toward an interaction of instruction and success (*F_(1,10)_* = 3.129, *p* = .082, MSE = .004).

## Discussion

This experiment used studied pairs presented under differing retrieval conditions to explore how parietal lobe subregions (Figure 2), default network (Figure 3), and posterior hippocampus (Figure 4) might be influenced by top-down processes directing attention to memory during retrieval success and failure. The findings suggest that the dorsal parietal region (BA 7) is minimally responsive to success and failure, but is greatly influenced by attention to memory. In contrast, ventral regions of the parietal lobe (BA 40 and 39) are highly sensitive to retrieval success, but only under conditions where retrieval is attempted. Directed suppression of retrieval abolishes any retrieval success effect in all three of the parietal regions. Default network was influenced by retrieval success, showing a larger decrease in activity for trials that were forgotten. Hippocampus was sensitive to retrieval success and attention to memory, suggesting that regional hippocampal activity can be influenced by top down processes directing attention toward or away from retrieval.

### Top-down and bottom-up modulation of parietal lobe activity

These findings inform discussion about the differential influences of top-down and bottom-up attentional processes on dorsal and ventral parietal subregions. Prior studies have suggested that ventral parietal activity is modulated by bottom-up capture of attention when a familiar stimulus is encountered, leading to a retrieval-success effect in the region [5]; however, the present findings demonstrate that this retrieval-success effect is abolished under conditions where subjects apply top-down suppression of retrieval. This would suggest that such top-down suppression affects a stage of stimulus processing that precedes bottom-up capture of attention. Though it is difficult to gauge the subjects' approaches to carrying out suppression of retrieval, the subjects were instructed not to close their eyes or look away as a strategy. Additionally, the continued presence of an impulse response function during the suppress condition suggests that at least some low-level processing of the stimuli took place.

Dorsal parietal cortex may be responsive to top-down direction of attention to memory, and the present results are consistent with this hypothesis. This region was less sensitive to retrieval-success effects and enhanced activity was seen for the elaborative encoding condition, a condition designed to maximize top-down attention directed toward retrieved material. Nevertheless, there was no difference in activity between suppressed trials and retrieved trials in this region, whereas attention to memory might be expected to be further reduced during suppressed trials. It remains possible that subjects performed the suppression task by directing attention to a distinctly different memory, and both retrieval and suppression are effortful processes involving direction of attention toward or away from a particular memory. One might then posit that effort and attention would be increased for suppression of stronger memories relative to suppression of weaker memories, but this was not supported through examination of brain activity in this study, as suppression of remembered items and suppression of forgotten items did not yield detectably distinct parietal lobe activity in any of the subregions. Still, some evidence in the literature exists for greater attention and BA 7 activity for invalid rather than valid cues [42], [43]. Thus, the present findings, together with findings from the literature, might alternatively support the idea that dorsal parietal regions are influenced by a change in direction of attention, including toward or away from memory, rather than simply by directing attention toward memory.

### Memory strength effects in the default network

Default network regions have shown retrieval-success or memory strength effects in a number of studies [4], [11], [44] and, in fact, note has been made about the high degree of overlap between the “retrieval network” and the default network [45]. The present results also reveal robust retrieval-success or memory strength effects in the default network, but shed further light on the bases of such effects by revealing that the primary modulator of such activity is further suppression of activity associated with forgotten responses. That is, the average beta values from across the default network demonstrate minimal modulation of activity by remembered responses and a high degree of modulation for forgotten responses. A possible explanation is that these regions have been shown to be modulated by task difficulty, and it seems reasonable to assume that retrieval tasks are more difficult when the target is forgotten. This finding helps inform previous studies on default network modulation during tasks of memory retrieval. A simple subtraction of retrieved minus forgotten trials would have shown “activation” in the default network of regions. However, when the directionality of response is examined it appears that this difference is due to greater amplitude of the task-negative response for forgotten trails and not due to task-positive activity for remembered trials.

This increased modulation of default network during forgotten responses compared to remembered responses could also be attributable to the process of episodic search. Forgotten pairs require more search than remembered pairs, leading to increasing retrieval effort with decreasing memory strength. Previous studies have shown the default network to be modulated by this increased retrieval effort [3], [31], [41] as well as episodic search [46], and, further, recent studies of episodic search processes during retrieval failures suggest that default network deactivations may be more directly linked to increased episodic search than to retrieval strength differences [47].

### Hippocampus

The present study is the first to demonstrate the dependence of hippocampal retrieval-success effects on top-down direction of attention toward or away from memory retrieval. Given the tight linkages between primary sensory cortices and hippocampal input structures [48] it is surprising that top-down processes can abolish such apparently early-level retrieval-success effects. Taken in conjunction with the findings in ventral parietal lobe, such hippocampal effects suggest the ability of directed attention to affect mnemonic processes at their earliest stages.

## Conclusion

By examining top-down influences on parietal and hippocampal brain activity linked to memory retrieval, this study highlights how attention-shifts, search, and post-retrieval processing are important drivers of such activity, in some cases even more than the retrieval event itself. This study found that top-down suppression affects early stimulus processing, possibly gating bottom-up capture of attention. Additionally, dorsal parietal cortex was found to be responsive to top-down attention to memory. This study adds to the body of literature suggesting functional heterogeneity of parietal lobe subregions during memory retrieval. Memory strength effects were found in the default network, and it was revealed that the primary modulator of these effects was further suppression of activity for forgotten responses. It was also found that hippocampal retrieval-success effects are dependent on the top-down direction of attention toward or away from memory retrieval. In this study, parietal lobe regions and hippocampus were defined anatomically. Finer-grained delineation within accepted anatomical regions could yield further functional dissociation even within a defined region [49]–[51]. Studying the interplay and dissociation of “ancillary” memory processes that include attention-shifts, search, and post-retrieval processing will provide a more complete explanation of the regional contributions to oft-described patterns of neuronal activity seen during memory retrieval.

## References

[1] Squire LR (2009) Memory and brain systems: 1969–2009. J Neurosci 29: 12711–12716. 19828780 10.1523/JNEUROSCI.3575-09.2009 PMC2791502

[2] Nelson SM, Cohen AL, Power JD, Wig GS, Miezin FM, et al (2010) A parcellation scheme for human left lateral parietal cortex. Neuron 67: 156–170. 20624599 10.1016/j.neuron.2010.05.025 PMC2913443

[3] Seibert TM, Gimbel SI, Hagler DJ, Brewer JB (2011) Parietal activity in episodic retrieval measured by fMRI and MEG. Neuroimage 55: 788–793. 21134473 10.1016/j.neuroimage.2010.11.078 PMC3035726

[4] Cabeza R (2008) Role of parietal regions in episodic memory retrieval: The dual attentional processes hypothesis. Neuropsychologia 46: 1813–1827. 18439631 10.1016/j.neuropsychologia.2008.03.019 PMC2517132

[5] Ciaramelli E, Grady CL, Moscovitch M (2008) Top-down and bottom-up attention to memory: a hypothesis (AtoM) on the role of the posterior parietal cortex in memory retrieval. Neuropsychologia 46: 1828–1851. 18471837 10.1016/j.neuropsychologia.2008.03.022

[6] Wagner AD, Shannon BJ, Kahn I, Buckner RL (2005) Parietal lobe contributions to episodic memory retrieval. Trends Cogn Sci 9: 445–453. 16054861 10.1016/j.tics.2005.07.001

[7] Henson RN, Rugg MD, Shallice T, Josephs O, Dolan RJ (1999) Recollection and familiarity in recognition memory: an event-related functional magnetic resonance imaging study. J Neurosci 19: 3962–3972. 10234026 10.1523/JNEUROSCI.19-10-03962.1999 PMC6782715

[8] Eldridge LL, Knowlton BJ, Furmanski CS, Bookheimer SY, Engel S a (2000) Remembering episodes: a selective role for the hippocampus during retrieval. Nat Neurosci 3: 1149–1152. 11036273 10.1038/80671

[9] Dobbins IG, Rice HJ, Wagner AD, Schacter DL (2003) Memory orientation and success: separable neurocognitive components underlying episodic recognition. Neuropsychologia 41: 318–333. 12457757 10.1016/s0028-3932(02)00164-1

[10] Wheeler ME, Buckner RL (2004) Functional-anatomic correlates of remembering and knowing. Neuroimage 21: 1337–1349. 15050559 10.1016/j.neuroimage.2003.11.001

[11] Vilberg KL, Rugg MD (2008) Memory retrieval and the parietal cortex: a review of evidence from a dual-process perspective. Neurobiology 46: 1787–1799. 10.1016/j.neuropsychologia.2008.01.004 PMC2488316 18343462

[12] Vilberg KL, Rugg MD (2009) Left parietal cortex is modulated by amount of recollected verbal information. Neuroreport 20: 1295–1299. 19668014 10.1097/WNR.0b013e3283306798 PMC2771458

[13] Shadlen MN, Newsome WT (2001) Neural Basis of a Perceptual Decision in the Parietal Cortex (Area LIP) of the Rhesus Monkey. J Neurophysiol 86: 1916–1936. 11600651 10.1152/jn.2001.86.4.1916

[14] Baddeley A (2000) The episodic buffer: a new component of working memory? Trends Cogn Sci 4: 417–423. 11058819 10.1016/s1364-6613(00)01538-2

[15] Ally BA, Simons JS, McKeever JD, Peers P V, Budson AE (2008) Parietal contributions to recollection: electrophysiological evidence from aging and patients with parietal lesions. Neuropsychologia 46: 1800–1812. 18402990 10.1016/j.neuropsychologia.2008.02.026 PMC2519009

[16] Olson IR, Berryhill M (2009) Some surprising findings on the involvement of the parietal lobe in human memory. Neurobiol Learn Mem 91: 155–165. 18848635 10.1016/j.nlm.2008.09.006 PMC2898273

[17] Okada K, Vilberg KL, Rugg MD (2011) Comparison of the neural correlates of retrieval success in tests of cued recall and recognition memory. Hum Brain Mapp 33: 523–533. 21455941 10.1002/hbm.21229 PMC3129402

[18] Hutchinson JB, Uncapher MR, Weiner KS, Bressler DW, Silver MA, et al (2012) Functional Heterogeneity in Posterior Parietal Cortex Across Attention and Episodic Memory Retrieval. Cereb Cortex 24: 49–66. 23019246 10.1093/cercor/bhs278 PMC3862264

[19] Daselaar SM, Fleck MS, Dobbins IG, Madden DJ, Cabeza R (2006) Effects of healthy aging on hippocampal and rhinal memory functions: an event-related fMRI study. Cereb Cortex 16: 1771–1782. 16421332 10.1093/cercor/bhj112 PMC1810232

[20] Fleck MS, Daselaar SM, Dobbins IG, Cabeza R (2006) Role of prefrontal and anterior cingulate regions in decision-making processes shared by memory and nonmemory tasks. Cereb Cortex 16: 1623–1630. 16400154 10.1093/cercor/bhj097

[21] Kim H, Cabeza R (2009) Common and specific brain regions in high- versus low-confidence recognition memory. Brain Res 1282: 103–113. 19501072 10.1016/j.brainres.2009.05.080 PMC2709704

[22] Herron JE, Wilding EL (2004) An electrophysiological dissociation of retrieval mode and retrieval orientation. Neuroimage 22: 1554–1562. 15275912 10.1016/j.neuroimage.2004.04.011

[23] Raichle ME, MacLeod AM, Snyder AZ, Powers WJ, Gusnard DA, et al (2001) A default mode of brain function. Proc Natl Acad Sci U S A 98: 676–682. 11209064 10.1073/pnas.98.2.676 PMC14647

[24] Buckner RL, Vincent JL (2007) Unrest at rest: default activity and spontaneous network correlations. Neuroimage 37: 1091–6 discussion 1097–1099. 17368915 10.1016/j.neuroimage.2007.01.010

[25] Mason M, Norton M, Horn J Van, Wegner D (2007) Wandering minds: the default network and stimulus-independent thought. Science (80-) 315: 393–395. 10.1126/science.1131295 PMC1821121 17234951

[26] Gusnard DA, Akbudak E, Shulman GL, Raichle ME (2001) Medial prefrontal cortex and self-referential mental activity: relation to a default mode of brain function. Proc Natl Acad Sci U S A 98: 4259–4264. 11259662 10.1073/pnas.071043098 PMC31213

[27] McKiernan KA, Kaufman JN, Kucera-Thompson J, Binder JR (2003) A parametric manipulation of factors affecting task-induced deactivation in functional neuroimaging. J Cogn Neurosci 15: 394–408. 12729491 10.1162/089892903321593117

[28] Esposito F, Bertolino A, Scarabino T, Latorre V, Blasi G, et al (2006) Independent component model of the default-mode brain function: Assessing the impact of active thinking. Brain Res Bull 70: 263–269. 17027761 10.1016/j.brainresbull.2006.06.012

[29] Fransson P (2006) How default is the default mode of brain function? Further evidence from intrinsic BOLD signal fluctuations. Neuropsychologia 44: 2836–2845. 16879844 10.1016/j.neuropsychologia.2006.06.017

[30] Stark CE, Squire LR (2001) When zero is not zero: the problem of ambiguous baseline conditions in fMRI. Proc Natl Acad Sci U S A 98: 12760–12766. 11592989 10.1073/pnas.221462998 PMC60127

[31] Gimbel SI, Brewer JB (2011) Reaction time, memory strength, and fMRI activity during memory retrieval: Hippocampus and default network are differentially responsive during recollection and familiarity judgments. Cogn Neurosci 2: 19–23. 21278912 10.1080/17588928.2010.513770 PMC3026441

[32] Rossion B, Pourtois G (2004) Revisiting Snodgrass and Vanderwart's object pictorial set: The role of surface detail in basic-level object recognition. Perception 33: 217–236. 15109163 10.1068/p5117

[33] Dale A (1999) Optimal experimental design for event-related fMRI. Hum Brain Mapp 8: 109–114. 10524601 10.1002/(SICI)1097-0193(1999)8:2/3<109::AID-HBM7>3.0.CO;2-W PMC6873302

[34] Brewer JB, Moghekar A (2002) Imaging the medial temporal lobe: exploring new dimensions. Trends Cogn Sci 6: 217–223. 11983585 10.1016/s1364-6613(02)01881-8

[35] Smith SM, Jenkinson M, Woolrich MW, Beckmann CF, Behrens TEJ, et al (2004) Advances in functional and structural MR image analysis and implementation as FSL. Neuroimage 23 Suppl 1: S208–219. 15501092 10.1016/j.neuroimage.2004.07.051

[36] Talairach J, Tournoux P (1988) A co-planar stereotaxic atlas of the human brain. Stuttgart, Germany: Thieme.

[37] Cox RW (1996) AFNI: software for analysis and visualization of functional magnetic resonance neuroimages. Comput Biomed Res 29: 162–173. 8812068 10.1006/cbmr.1996.0014

[38] Miller MI, Beg MF, Ceritoglu C, Stark C (2005) Increasing the power of functional maps of the medial temporal lobe by using large deformation diffeomorphic metric mapping. Proc Natl Acad Sci U S A 102: 9685–9690. 15980148 10.1073/pnas.0503892102 PMC1172268

[39] Insausti R, Juottonen K, Soininen H, Insausti a M, Partanen K, et al (1998) MR volumetric analysis of the human entorhinal, perirhinal, and temporopolar cortices. AJNR Am J Neuroradiol 19: 659–671. 9576651 PMC8337393

[40] Kirwan CB, Jones C, Miller M, Stark CEL (2007) High resolution fMRI investigation of the medial temporal lobe. Hum Brain Mapp 28: 959–966. 17133381 10.1002/hbm.20331 PMC2853185

[41] Israel SL, Seibert TM, Black ML, Brewer JB (2010) Going their separate ways: dissociation of hippocampal and dorsolateral prefrontal activation during episodic retrieval and post-retrieval processing. J Cogn Neurosci 22: 513–525. 19301994 10.1162/jocn.2009.21198

[42] O'Connor AR, Han S, Dobbins IG (2010) The inferior parietal lobule and recognition memory: expectancy violation or successful retrieval? J Neurosci 30: 2924–2934. 20181590 10.1523/JNEUROSCI.4225-09.2010 PMC2844718

[43] Jaeger A, Konkel A, Dobbins IG (2013) Unexpected novelty and familiarity orienting responses in lateral parietal cortex during recognition judgment. Neuropsychologia 51: 1061–1076. 23499719 10.1016/j.neuropsychologia.2013.02.018 PMC3690975

[44] Daselaar SM, Prince SE, Dennis NA, Hayes SM, Kim H, et al (2009) Posterior midline and ventral parietal activity is associated with retrieval success and encoding failure. Front Hum Neurosci 3: 13–23. 19680466 10.3389/neuro.09.013.2009 PMC2726033

[45] Buckner RL, Andrews-Hanna JR, Schacter DL (2008) The brain's default network: anatomy, function, and relevance to disease. Ann N Y Acad Sci 1124: 1–38. 18400922 10.1196/annals.1440.011

[46] Reas ET, Gimbel SI, Hales JB, Brewer JB (2011) Search-Related Suppression of Hippocampus and Default Network Activity during Associative Memory Retrieval. Front Hum Neurosci 5: 1–13. 22046159 10.3389/fnhum.2011.00112 PMC3202230

[47] Reas ET, Brewer JB (2013) Retrieval search and strength evoke dissociable brain activity during episodic memory recall. J Cogn Neurosci 25: 219–233. 23190328 10.1162/jocn_a_00335 PMC4091972

[48] Suzuki Wa, Amaral DG (2003) Perirhinal and parahippocampal cortices of the macaque monkey: cytoarchitectonic and chemoarchitectonic organization. J Comp Neurol 463: 67–91. 12811804 10.1002/cne.10744

[49] Cavanna AE, Trimble MR (2006) The precuneus: a review of its functional anatomy and behavioural correlates. Brain 129: 564–583. 16399806 10.1093/brain/awl004

[50] Huijbers W, Schultz AP, Vannini P, McLaren DG, Wigman SE, et al (2013) The encoding/retrieval flip: interactions between memory performance and memory stage and relationship to intrinsic cortical networks. J Cogn Neurosci 25: 1163–1179. 23384193 10.1162/jocn_a_00366 PMC3730829

[51] Cabeza R, Ciaramelli E, Moscovitch M (2012) Cognitive contributions of the ventral parietal cortex: an integrative theoretical account. Trends Cogn Sci 16: 338–352. 22609315 10.1016/j.tics.2012.04.008 PMC3367024

